# Daily cycling of nitric oxide synthase (NOS) in the hippocampus of pigeons (*C. livia*)

**DOI:** 10.1186/1740-3391-11-12

**Published:** 2013-11-01

**Authors:** Aline V Machado-Nils, Larissa OM de Faria, André S Vieira, Simone A Teixeira, Marcelo N Muscará, Elenice AM Ferrari

**Affiliations:** 1 Departamento de Biologia Estrutural e Funcional, Instituto de Biologia, Universidade Estadual de Campinas (UNICAMP), Rua Monteiro Lobato, 255, Campinas, São Paulo 13083-970, Brazil; 2 Departamento de Farmacologia, Instituto de Ciências Biomédicas, Universidade de São Paulo (USP), São Paulo, SP, Brazil

**Keywords:** Nitric oxide, Neuronal nitric oxide synthase (nNOS), Hippocampus, Pigeon, Daily molecular cycling

## Abstract

**Background:**

Nitric oxide synthase (NOS) is essential for the synthesis of nitric oxide (NO), a non-conventional neurotransmitter with an important role in synaptic plasticity underlying processes of hippocampus-dependent memory and in the regulation of biological clocks and circadian rhythms. Many studies have shown that both the NOS cytosolic protein content and its enzymatic activity present a circadian variation in different regions of the rodent brain, including the hippocampus. The present study investigated the daily variation of NOS enzymatic activity and the cytosolic content of nNOS in the hippocampus of pigeons.

**Results:**

Adult pigeons kept under a skeleton photoperiod were assigned to six different groups. Homogenates of the hippocampus obtained at six different times-of-day were used for NOS analyses. Both iNOS activity and nNOS cytosolic protein concentrations were highest during the subjective light phase and lowest in the subjective dark phase of the circadian period. ANOVA showed significant time differences for iNOS enzymatic activity (p < 0.05) and for nNOS protein content (p < 0.05) in the hippocampus. A significant daily rhythm for both iNOS and nNOS was confirmed by analysis with the Cosinor method (p < 0.05). The present findings indicate that the enzymatic activity of iNOS and content of nNOS protein in the hippocampus of pigeons exhibit a daily rhythm, with acrophase values occurring during the behavioral activity phase.

**Conclusions:**

The data corroborate the reports on circadian variation of NOS in the mammalian hippocampus and can be considered indicative of a dynamic interaction between hippocampus-dependent processes and circadian clock mechanisms.

## Background

Nitric oxide synthase (NOS) plays an essential role in the synthesis of nitric oxide (NO) which has an important role as a mediator in many physiological processes, including mechanisms regulating biological clocks and circadian rhythms [1]. NO synthesis results from the oxidation of L-arginine by the enzyme NO synthase (NOS) which is found as one of three main types. Two are constitutive nitric oxide synthases (cNOS): endothelial nitric oxide synthase (eNOS) and neuronal nitric oxide synthase (nNOS) and are dependent of Ca^2+^, and the other isoform is the inducible nitric oxide synthase (iNOS), which is independent of Ca^2+^[2]. The majority of the information available on the role of NO in the brain deals with nNOS, of which the brain contains the highest activity found in any tissue, and which, although present in some cerebral vessels and in glial cells, is predominantly found in neurons [2,3]. In the CNS, NO synthesis seems to be predominantly regulated by the influx of Ca^2+^ through glutamate receptor channels, in particular following postsynaptic stimulation of NMDA receptors [4-7].

Circadian variation of both NOS activity and cytosolic protein content has been reported in several regions of the brain of rodents, such as cerebellum, brainstem, hypothalamus and hippocampus, which showed acrophase in the dark phase [8]. Rhythmic expression of NOS was also observed in total brain tissue homogenate when both exposed to light/dark cycle and to constant light [9]. Evidence indicates that like in rodents, the hippocampus of birds also has cells containing NOS, which are involved in the formation and retrieval of memory [10,11]. Additionally, the hippocampus of the pigeon has a wide distribution of glutamate receptors [12] which can mediate the activation of NOS and NO synthesis and mechanisms of synaptic plasticity.

The diurnal oscillation of NOS in the hippocampus is similar to those described for molecules that participate in processes of consolidation and persistence of hippocampus-dependent memory in rodents [13-16], which are also initiated by Ca^2+^ intracellular signaling originating from glutamate NMDA receptors. Many studies have shown that drugs that inhibit nNOS activity can block hippocampus-dependent processes such as long-term potentiation (LTP) and long-term memory processes [17-19].

Although daily fluctuation of molecules in intracellular pathways has been described for circuits of the hippocampus in rodents [13-15], a 24 h profile of the NOS activity has not been studied in the avian hippocampus. So, the analysis of molecular cycling in the hippocampus of pigeons can add important points to the set of many studies that described a coordinated set of circadian biochemichal, physiological and behavioral rhythms in a wide array of vertebrates, including birds [20-23]. Particularly, pigeons have been much studied in laboratory research on circadian rhythms of feeding, thermal physiology, hormonal variation and metabolism [24-27] as well as on time-of-day variation of behavior and learning [28-31]. Therefore, the present study was performed to elucidate the 24 h cycling of NOS in the hippocampus of pigeons. The enzymatic activity of Ca^2+^-dependent (cNOS) and Ca^2+^-independent NOS (iNOS) and the expression of the nNOS protein were analyzed.

## Materials and methods

Forty-eight male pigeons (*Columba livia*) were used. For 15 days, these pigeons were housed in individual cages within an isolated room, under a 12:12 h light–dark cycle (light: 1000 lux; dark: 0 lux; lights on at 6:00 h) and temperature maintained at 22°C. After this period, the light cycle was gradually changed to a skeleton photoperiod, with two 15-min pulses of bright white light (1000 lux) separated from each other by 11:45 h of dim red light (5 lux).

During four days, the animals were taken from their cage and transported to the laboratory, where they were weighed before being returned to the cages. In the fifth day, the pigeons were divided into six different groups (n = 8): ZT0, ZT4, ZT8, ZT12, ZT16, ZT20. ZT0 corresponds to the time of turning on the light. The hippocampi of the pigeons were collected after decapitation conducted at six different times-of-day, one time for each group. After brain removal, the dissection of the hippocampus was performed according to the coordinates of the atlas of Karten and Hodos [32]. The tissue was immediately frozen in liquid nitrogen and subsequently stored at −80°C. All experimental procedures were conducted in accordance with the requirements of the Ethics Committee for Animal Experimentation of the Biology Institute, UNICAMP, Brazil, (Protocol 1732–1).

The enzymatic activity of cNOS [33] was analyzed in samples of hippocampus of pigeons pertaining to each group. The samples were homogenized in 5 V of cold incubation buffer (50 mM Tris–HCl buffer, pH 7.4) containing 1 mM phenylmethyl-sulphonyl fluoride (PMSF) and 1 mM L-citrulline. The homogenates were incubated for 30 min in the presence of 1 mM NADPH, 2 mM CaCl2 and 10 uM L-arginine containing 100.000 dpm of [2,3,4,5-H]L-arginine monohydrochloride (Amersham, UK) at room temperature (25–27°C). Pharmacological controls of enzymatic activity were also conducted without CaCl_2_ both to identify the type of NOS (addition of 1 mM EGTA, a Ca^2+^ influx inhibitor) and to selectively inhibit NOS (addition of 1 mM L-NAME). Protein content of the samples was determined by the Bradford Method [34] using a commercial kit (Bio Rad, Hercules, CA, USA). NOS activity was expressed as pmols L-citrulline produced per minute and per milligram of protein.

Protein expression of nNOS was also analyzed in homogenates of the hippocampus by the Western blot method using four pigeons per group. For total protein quantification, samples were homogenized in 1% Triton X-100, 50 mM phosphate buffer, pH 7.4, 1 mM sodium pyrophosphate, 1 mM sodium fluoride, 5 mM EDTA, 1 mM sodium vanadate, 1% protease inhibitor cocktail (P8340; Sigma), 7 M urea, and 2 M thiourea (10% w/v). Sample homogenization was carried out at 4°C using a Polytron 20 s generator set at maximum speed for 30 s. Insoluble materials were removed by centrifugation (12 000 g, 4°C, 15 min). Protein concentration was determined using the Bradford method [34]. One hundred milligrams of total protein extract from each animal was separated by SDS–polyacrylamide gel electrophoresis and electroblotted to a nitrocellulose membrane [35,36]. Membranes were blocked with PBS–Tween containing 5% non-fat dry milk and then incubated with a rabbit polyclonal antibody to nNOS (NOS1 (R-20): sc 648, Santa Cruz Biotechnology, Santa Cruz, CA, USA) diluted (1:1000) in PBS–Tween containing 3% bovine serum albumin (12 h at 4°C). Membranes were washed with PBS–Tween and incubated with horseradish peroxidase–conjugated goat antibody to rabbit (1 : 10 000; Zymax, Zymed Laboratories, USA). The immunoreactive bands were detected by autoradiography on a Kodak GBX2 film using a SuperSignal West Pico chemiluminescent kit (Pierce, Rockford, IL, USA). Equal protein loading was assessed with Ponceau S staining of the membranes and optical density analysis of the various protein bands [37]. The optical density of the immunoreactive bands was determined by digital densitometry (Scion Image Software).

The enzymatic activity of Ca^2+^-dependent NOS and Ca^2+^-independent and optical densitometry data furnished by Western blot for nNOS expression were adjusted to a cosine curve [38] with a 24-hour period [39]. The data were analyzed using a one-way ANOVA, considering time as variable. The Tukey-Kramer test was used for *post-hoc* multiple comparisons.

## Results

**Figure 1:**
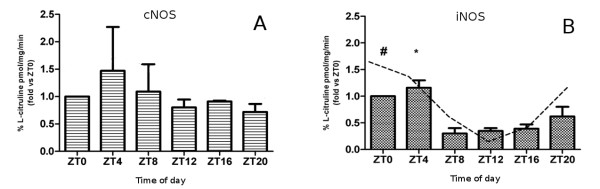
**Enzymatic activity of cNOS and iNOS in hippocampus of pigeon. A**- Mean values (+ s.e.m.) of the amount of L-citrulline (pmol/min/mg) produced by the reaction of the enzymatic activity of Ca^2+^ - dependent NOS (cNOS) in samples of hippocampal tissue homogenate. Data are expressed as ratio calculated relative to the mean of ZT0 group. One-way ANOVA. **B**- Mean values (+ s.e.m.) in the amount of L-citrulline (pmol/min/mg) produced by the reaction of the enzymatic activity of Ca^2+^ - independent NOS (iNOS) in samples of hippocampal tissue homogenate expressed as ratio relative to the mean of ZT0 group. One-way ANOVA indicated between group differences *(p < 0.001);* * indicates *p* < 0.05 when compared to ZT8, ZT12 and ZT16; # indicates *p* < 0.05 compared with ZT12 and (+ s.e.m.) ZT16 (Tukey-Kramer test; n = 4 pigeons per group).

Figure 1 shows the enzymatic activity of cNOS (Figure 1A) and iNOS (Figure 1B) values for groups ZT0, ZT4, ZT8, ZT12, ZT16 and ZT20 in the hippocampus. Enzymatic activity was measured as the amount of L-citrulline produced in the reaction between NOS and L-arginine per minute and milligram of protein. The mean values for the absolute activity of cNOS were 0.65 ± 0.38 (ZT0); 0.96 ± 0.52 (ZT4); 0.71 ± 0.37 (ZT8); 0.52 ± 0.10 (ZT12); 0.59 ± 0.01 (ZT16) and 0.47 ± 0.22 (ZT20) and for iNOS the mean values were 0.83 ± 0.30 (ZT0); 0.95 ± 0.18 (ZT4); 0.16 ± 0.10 (ZT8); 0.33 ± 0.07 (ZT12); 0.25 ± 0.22 (ZT16); 0.47 ± 0.21 (ZT20). Figure 1A and 1B present data expressed as the ratio between the mean value of each group and the mean value of the ZT0 group. Statistical analysis with one-way ANOVA showed significant time differences for iNOS (F_(5,18)_ = 5.93; p < 0.05) but not for cNOS (F_(5,18)_ = 0.27; p > 0.05). Tukey-Kramer comparisons test showed that the ZT0 and the ZT4 groups had values of iNOS enzymatic activity that differed significantly from the ZT8, ZT12 and ZT16 groups (p < 0.05).

**Figure 2:**
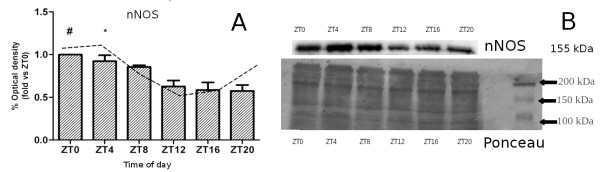
**Protein expression of nNOS in hippocampus of pigeon. A**- Mean values (+ s.e.m.) of optical densitometric analyses of immunoreactive bands for nNOS in samples of hippocampal tissue homogenate normalized with Ponceau staining. Each mean was calculated with reference to the ZT0 value. The dashed line indicates the fitted curve by the Cosinor method. One-way ANOVA indicated group effects (*p <* 0.001); * indicates *p* < 0.05 when compared to ZT12, ZT16 and ZT20; # indicates *p* < 0.05 compared with ZT16 and ZT20 (Turkey-Kramer test; n = 4 pigeons per group). **B**- Representative immunoreactive bands for nNOS and proteins detected by Ponceau staining in each group.

Optical densitometry values of the nNOS immunoreactive bands (Figure 2A) were normalized for the total protein content of the samples as determined by Ponceau S solution for histochemical staining (Figure 2B). ANOVA indicated significant differences between groups (F_(5,18)_ = 7.6; p < 0.05). Tukey-Kramer test showed that the ZT0 group differed significantly from the ZT12, ZT16 and ZT20 groups whereas the ZT4 group was significantly different from the ZT16 and ZT20 groups (p < 0.05).

Table 1 presents data on the rhythmic characteristics of iNOS enzymatic activity and nNOS protein content in the hippocampus that were obtained with the 24-hour Cosine Curve fit method (Cosana software [39]). The percent of rhythmic values obtained with the cosine curve analysis indicated oscillation of nNOS protein expression in the hippocampus. In addition, the cosine analysis also indicated oscillation of enzymatic activity of iNOS. The values of enzymatic activity of iNOS and nNOS protein content in the hippocampus showed significant rhythmicity (p < 0.05; Cosinor test).

**Table 1:** Parameters of the circadian rhythmicity in the enzymatic activity of iNOS and of nNOS protein expression, obtained by adjusting the cosine curve by method Cosinor (Benedito-Silva, 1988)

	**Acrophase**	**Bathyphase**	**Amplitude**	**Mesor**	**%R**	***P***
**iNOS activity**	6 h36 a.m. ± 94 min	6 h36 p.m. ± 94 min	0,46 ± 0,19	0,29	52,10	0,001
**nNOS content**	9 h50 a.m. ± 38 min	9 h50 p.m. ± 38 min	0,24 ± 0,07	0,77	67,57	0,01

R% - Percent Rhythmic.

Acrophase - phase in which it is more likely to find the highest value of a sine curve set.

Mesor - average value of the fitted curve by the method Cosinor.

## Discussion

The present results show that circadian oscillation of the nNOS protein expression and of the enzymatic activity of iNOS occurs in the hippocampus of pigeon. The oscillation of nNOS protein expression was more robust than that observed for iNOS activity as shown by higher percent rhythmic value for nNOS content than the percent rhythmic value for iNOS. The analysis of enzymatic activity values of cNOS did not show significant statistical differences between different time-points. However, the analysis with the cosine curve fit was indicative of daily oscillation of cNOS activity. We may conjecture that the statistical significance of the data was limited by the inter-individuals variability of the cNOS activity that was mainly observed at the time points during the subjective day, although the peak of cNOS enzymatic activity observed at ZT04 may have induced a detection of oscillation by the cosine analysis. The Cosinor procedure is considered to be adequate for detection of rhythmicity of molecular circadian oscillation in typical studies that use groups as small as 3 animals [40]. Our study used groups of 4 pigeons, but it can be considered that the use of more animals per group would improve the validity of the data. It is worth to note that the analysis of the activity of cNOS considers the activity of both eNOS and nNOS isoforms, whereas the analysis for the protein content refers only to nNOS. Thus, in each case the resulting rhythmicity was different, as demonstrated by the significant daily cycling in the hippocampus of pigeon only for expression of nNOS. In spite of this, we may consider that our results agree with the experimental evidence showing that the peak of NOS activity in the hippocampus and other brain regions of rats occurs during the activity phase [8,9]. Additionally, since the nNOS protein accounts for the major part of NO production in the mammalian brain [2,3], the present data can be considered as indirect evidence of circadian rhythm of NO signaling in the hippocampus of the pigeon.

The acrophase value of nNOS protein content occurred 9:50 a.m. ± 38 min in the hippocampus with a peak value around ZT4, during the activity phase and a trough value at ZT20, during the resting phase. Daily variation of NOS activity and protein levels have been described in the rat brain [8] and recent evidence showed that the oscillation of adenylyl ciclase and MAPK in the hippocampus is necessary for maintenance of hippocampal-dependent memory [15,16]. The repeated reactivation of NMDA receptors and intracellular Ca^2+^ signaling leads to activation of the cAMP-MAPK-CREB pathway [13-16] which has a central role in memory processes [15]. So, oscillations of NOS in the hippocampus may also contribute to processes of consolidation and maintenance of hippocampus-dependent memories.

In fact, nNOS activity in the central nervous system correlates with activation of NMDA receptors [41], which are widely localized in the hippocampus of both rodents [42] and pigeons [12]. Synaptic processes mediated by glutamate also triggers the activation of a signal transduction pathway which involves Glu- Ca^2+^/calmodulin-dependent kinase (Ca^2+^-CaMKII)-nNOS-GC-cGMP and clock genes transduction. Pharmacological inhibition of the nNOS or the cGMP-dependent kinase blocks the circadian responses to light in vivo [1]. Previous studies have demonstrated that processes related to cellular signaling involving nNOS induce changes in transcription of clock genes in the suprachiasmatic nucleus of rodents and are also involved with processes of synchronization and phase shift [1,43,44]. These results suggest a role of nNOS, and consequently NO, in the regulation of biological oscillators. However, it has yet to be determined how the expression of clock genes contributes to the maintenance of NOS oscillations in the hippocampus or vice-versa.

In addition, melatonin receptors in the hippocampus are coupled to inhibitory protein G [45,46] and the inhibition of Ca^2+^ -mediated mechanisms by nocturnal melatonin could explain NOS oscillation in the hippocampus. Accordingly, lesion in the SCN blocked the circadian oscillation of Ca^2+^ stimulated adenylyl ciclase and MAPK activities in the hippocampus of mice, suggesting that the SCN function is required for the molecular circadian oscillation in the hippocampus [16]. These findings indicate that the SCN can indirectly modulate Ca^2+^ stimulated adenylyl ciclase in the hippocampus during the circadian cycle by controlling the release of melatonin from the pineal gland, which is a major efferent pathway of the biological timing system [47]. The expression and activity of both nNOS and iNOS proteins may be dependent on circadian timing system and according to recent evidence melatonin may be involved in the regulation of these mechanisms [48-50]. A transient but substantial rise of the constitutive nNOS was observed when cultured cells were incubated during 6 hours with 1 nM melatonin volume [50]. On the other hand, high plasmatic melatonin concentration was shown to inhibit the expression of both nNOS and iNOS [48,49,51,52]. In birds, both pineal and retinal melatonin have also an important role in the control of avian circadian rhythms [20,23]. A robust light–dark rhythm of melatonin was detected in pigeons, with low plasma melatonin in the light phase. The nocturnal peak was observed at 03:00 h with values 100–300 pg/ml of circulating melatonin [53]. In the present study, the peak of nNOS expression was observed around 10 a.m., that is, it occurred around seven hours after the peak of melatonin reported by the previous study on melatonin rhythm in pigeons conducted in our laboratory [53]. So, these results are consistent with the findings on the temporal expression of nNOS mRNA in *in vitro* study with human cells [50].

These findings in diurnal animals make intriguing relationship of melatonin and NOS in nocturnal animals, since the pace of both NOS activity and melatonin in rodents have peaks during the dark phase [8,9,54,55]. The experimental evidence collected in mammals showing cNOS diurnal oscillation is scarce and related with nocturnal rodents, as is seen in the studies reported by Ayers *et al.*[8] and Tuçtan *et al.*[9]. Besides, to the extent of our knowledge, our results provide the first description of daily rhythmicity of nNOS protein content in the hippocampus of the pigeon. So, more investigations are still needed for comparisons of the phase relationship of the curves of melatonin and NOS in other diurnal and nocturnal species. Nevertheless, it is reasonable to consider the occurrence of the peak of nNOS protein content in the hippocampus during the light phase of a diurnal animal, such as the pigeon, since during the activity phase of diurnal animals there is a requirement for high processing of environmental information that results in learning and memory, and, additionally, these processes are fundamentally related with the nNOS activity in the brain. Accordingly, it may not be surprising that nocturnal and diurnal animals exhibit daily oscillation of biological variables with peaks occurring at different circadian phases*.* In this sense, it may reasonable to consider that this fact can also be related with the higher variability of cNOS enzymatic activity during the subjective day.

Different NOS isoforms are considered to play distinct roles in the CNS. The nNOS isoform has been pointed as the major NOS isoform that is essentially involved with enhancement of memory formation and consolidation [56,57], although eNOS has been also indicated to participate in processes of memory formation [58]. Although the nNOS activity in the hippocampus is a key factor for learning and memory processes, Rappanelli *et al.*[59] showed that both eNOS and nNOS activities in the prefrontal cortex and hippocampus were augmented during the operant learning process in rats. Besides, exposures to uncontrollable or severe stressors induced nNOS expression in brain structures including the hippocampus, amygdala and cortex [59] and there is evidence indicating that increased production of NO in the dorsolateral periaqueductal gray area is involved in the anxiety behavior displayed by rats [60]. Additionally, both nNOS and iNOS participate of mechanisms related with neurogenesis in the hippocampus, since decreases in nNOS activity or increases in iNOS expression have been reported to promote neurogenesis in the dentate gyrus [57-60]. Besides, it is assumed that there is an inverse relation between increases in the systemic expression of iNOS and the amount of encephalic nNOS [61], although the mechanism regulating this relation still needs better comprehension. Though iNOS has been noted for its role in the brain defense mechanisms and deleterious processes, there are evidence of increased expression of iNOS in the hippocampus after training in spatial memory tasks [62,63], which draws attention to its possible role in mechanisms of learning and memory formation [63].

Therefore, the present data demonstrate that an oscillation in iNOS activity and nNOS protein content occurs in the hippocampus of pigeons. These observations extend the evidence of molecular cycling in the hippocampus to the avian hippocampus and may be seen as directly related to reports on the relationship between the circadian system and the molecular mechanisms of learning and memory [64,65]. Furthermore, these observations extend the knowledge on the similarities between the neurochemical, intrinsic and extrinsic organizational and functional characteristics of the avian and mammalian hippocampus [12,66-68].

## Competing interests

The authors declare that there is no conflict of interest that may be perceived as prejudicing the impartiality of the research reported.

## Authors’ contributions

AVMN conceived of the study, and participated in its design, carried out the western blot, enzymatic activity, statistical analysis and wrote the manuscript. LOMF contributed with data analysis and drafted the manuscript. ASV carried out the western blot. SAF carried out the enzymatic activity. MNM contributed with laboratory conditions for enzymatic analysis. EAMF conceived of the study, its design, and coordination and participate in the manuscript writing. All authors read and approved the final manuscript.
